# Chikungunya outbreak (2015) in the Colombian Caribbean: Latent classes and gender differences in virus infection

**DOI:** 10.1371/journal.pntd.0008281

**Published:** 2020-06-03

**Authors:** Oscar M. Vidal, Jorge Acosta-Reyes, Jesús Padilla, Edgar Navarro-Lechuga, Elsa Bravo, Diego Viasus, Mauricio Arcos-Burgos, Jorge I. Vélez

**Affiliations:** 1 Universidad del Norte, Barranquilla, Colombia; 2 Epidemiological Surveillance Team, Health Secretary Program, Barranquilla, Colombia; 3 Grupo de Investigación en Psiquiatría (GIPSI), Departamento de Psiquiatría, Instituto de Investigaciones Médicas, Facultad de Medicina, Universidad de Antioquia, Medellín, Colombia; Mahidol University, THAILAND

## Abstract

Chikungunya virus (CHIKV), a mosquito-borne alphavirus of the *Togaviridae* family, is part of a group of emergent diseases, including arbovirus, constituting an increasing public health problem in tropical areas worldwide. CHIKV causes a severe and debilitating disease with high morbidity. The first Colombian autochthonous case was reported in the Colombian Caribbean region in September 2014. Within the next two to three months, the CHIKV outbreak reached its peak. Although the CHIKV pattern of clinical symptomatology has been documented in different epidemiological studies, understanding of the relationship between clinical symptomatology and variation in phenotypic response to CHIKV infection in humans remains limited. We performed a cross sectional study following 1160 individuals clinically diagnosed with CHIKV at the peak of the Chikungunya outbreak in the Colombian Caribbean region. We examined the relationship between symptomatology and diverse phenotypic responses. Latent Class Cluster Analysis (LCCA) models were used to characterize patients’ symptomatology and further identify subgroups of individuals with differential phenotypic response. We found that most individuals presented fever (94.4%), headache (73.28%) and general discomfort (59.4%), which are distinct clinical symptoms of a viral infection. Furthermore, 11/26 (43.2%) of the categorized symptoms were more frequent in women than in men. LCCA disclosed seven distinctive phenotypic response profiles in this population of CHIKV infected individuals. Interestingly, 282 (24.3%) individuals exhibited a lower symptomatic “extreme” phenotype and 74 (6.4%) patients were within the severe complex “extreme” phenotype. Although clinical symptomatology may be diverse, there are distinct symptoms or group of symptoms that can be correlated with differential phenotypic response and perhaps susceptibility to CHIKV infection, especially in the female population. This suggests that, comparatively to men, women are a CHIKV at-risk population. Further study is needed to validate these results and determine whether the distinct LCCA profiles are a result of the immune response or a mixture of genetic, lifestyle and environmental factors. Our findings could contribute to the development of machine learning approaches to characterizing CHIKV infection in other populations. Preliminary results have shown prediction models achieving up to 92% accuracy overall, with substantial sensitivity, specificity and accuracy values per LCCA-derived cluster.

## Author summary

The Chikungunya virus (CHIKV) infection is a mosquito-borne virus of the *Togaviridae* family, part of the arbovirus group of mosquito-transmitted pathogens. CHIKV causes a severe and debilitating disease with high morbidity. In this study, we comprehensively analysed clinical data from 1160 individuals from the Colombian Caribbean, who were diagnosed with CHIKV infection during the 2014 epidemic peak and before the Zika epidemic (registered back in 2015). Further, the presence of latent classes and predictors of CHIKV susceptibility and severity of the CHIKV infection were analysed. Although it is well known that people respond differently to infection, our results showed that these differences are not arbitrary and may come from the specific orchestration of our immune response and specific genetic makeup. For example, we identified that females infected with CHIKV exhibited significant and heterogeneous phenotypic response patterns compared to men. Overall, these results inform about potential predictors and outlining strategies to study the natural history of CHIKV infection. Future studies assessing the contribution of demographic, immunological and genetic factors to symptom co-occurrence could shed some light on the severity of the clinical symptomatology and, ultimately, lead to more accurate, more efficient and differential diagnosis. These results could contribute to the development of machine learning approaches to characterizing CHIKV infection in other populations and provide more accurate and differential diagnosis.

## Introduction

Chikungunya virus (CHIKV) is a positive sense single-stranded RNA alphavirus, primarily transmitted by vectors such as mosquitoes, *Aedes aegypti* and *Aedes albopictus*.[1,2] Chikungunya’s name derives from the Bantu language (Makonde ethnic group from Tanzania and Mozambique) where CHIKV was first described in 1952 and refers to a body posture showing inability to walk and debilitating joint pain.[3,4] Estimates indicate that ~2 million people were reported to be infected by CHIKV during outbreaks in the 2000s.[5]

The first autochthonous CHIKV epidemic in America and the Caribbean was reported on the island of Saint Martin at the end of 2013.[2] The Asian and Eastern/Central/Southern African CHIKV strains were the agents causing the epidemic outbreak.[3] In September 2014, the Colombian health surveillance authorities reported the first autochthonous CHIKV case. Three months later, the Colombian Caribbean region reported a generalized CHIKV outbreak.[6]

The CHIKV outbreaks dramatically increased in areas with no prior immunity. Indeed, between 10%-70% of the population became infected and half of the infected population developed severe clinical symptomatology,[1,7] that is, febrile temperature above 38.9°C, polyarthralgia, maculopapular rash, rigor, myalgia, headache, photophobia, polyarthritis, and myositis.[8] The incubation period may last between 3 to 7 days. After viral inoculation by the mosquito, the virus replicates in the skin and then spreads, via the blood stream, to the liver and joints.[9] This acute phase lasts between 1–12 days reaching viral loads of 10^5^−10^12^ RNA copies per milliliter of blood.[9,10]

Several studies reported variable chikungunya symptomatology, i.e., La Reunion outbreak in 2004 showed patients with asymmetrical bilateral polyarthralgia (96%) of the lower (98%) and small joints (75%), asthenia (89%), headache (70%) myalgia (59%) and adenopathy (9%).[3,11] The 2006 CHIKV outbreak in India reported more that 1.4 million people affected whose clinical manifestations included joint pain, headache, abdominal pain, joint swelling, vomiting, rash, and cough.[12] Ray et al. (2012) reported different joint pain comorbidity in two Indian cities (i.e., Delhi and Karnataka) with heterogenous incidences of 40% and 85%, respectively.[13] CHIKV symptoms reported in other epidemics, such as diarrhea, rhinitis, conjunctival congestion, hepatomegaly, splenomegaly and lymphadenopathy were rarely observed in the India outbreak.[13,14] Comparison of acute clinical symptoms between CHIKV outbreaks in La Reunion, India, Singapore, Malaysia, and other Indian Ocean islands exhibited typical clinic, with fever, arthralgia, headache, and joint compromise (epidemiology data reported high percentages, between 80% to 100%).[14,15]

Global climate change, increases in tourism and international mobilization may contribute to the emergence or re-emergence of the alphaviruses associated with polyarthritis/arthralgia, such as: Sindbis-group viruses, Scandinavian Ockelbo virus, the African O’nyong-nyong virus (ONN), the South American Mayaro virus (MAY), the Australian Barmah Forest virus (BFV), Ross River virus (RRV) and the African/Asian Chikungunya virus (CHIK)[16–20]. Among these, the RRV and CHIKV have shown similar epidemiological features, such as polyarthritis, fever and rash, as CHIKV spread in the Americas in 2014, the RRV spread to the South Pacific, causing large epidemics in Fiji, the Cook Islands, and Samoa in 1979 [21,22].

Epidemiology reports show fluctuating typical and atypical symptoms from one epidemic to another, possibly due to: *i*) specific environment, *ii*) the genetic and immunological makeup of the infected population, *iii*) the viral strain and *iv*) the specific applied epidemiological models. This clinical heterogeneity reported across epidemiological and clinical studies of CHIKV outbreaks highlights the need for and importance of a rigorous clinical profile of the CHIKV infection in humans.[8,23,24]

In this study, we comprehensively analyzed clinical data from 1160 individuals from the metropolitan area of Barranquilla, Colombia, located on the northern Caribbean coast. Patients were diagnosed with CHIKV infection to evaluate the presence of group of individuals clustering similar and unique phenotypes (latent classes), predictors of infection susceptibility, and differential phenotypic response, i.e., a set of specific symptoms that an individual generates in response to CHIKV infection. We found strong evidence for the existence of different mutually exclusive profiles of phenotypic response in this population, and that females infected with CHIKV exhibited significant and heterogeneous differential symptomatology patterns when compared to men. For the first time, these results offer information about the characteristics of at-risk populations affected by CHIKV infection and the presence of different subpopulations of phenotypic response. Although future studies are needed to better understand the contribution of demographic, immunological and genetic factors to this differential phenotypic response, especially in this understudied population, our findings could be used as a starting point for the development of machine learning approaches to characterizing CHIKV infection in other populations, in order to provide more accurate and differential diagnosis and treatment.

## Subjects and methods

### Study design, target population and data collection

A cross-sectional analysis of patients clinically diagnosed with CHIKV, ascertained through the “*Programa de Vigilancia Epidemiológica de la Secretaría de Salud”* (*Health Secretary Program of Epidemiological Surveillance*) in Barranquilla, Colombia, was performed to evaluate the presence of distinct subgroups of patients according to their clinical profiles. This program is responsible for surveying and reporting outbreaks of infectious diseases occurring in this geographical area of the Colombian Caribbean coast. World Health Organization (WHO) recommendations were followed to recruit CHIKV infected patients and samples.[25]

**Fig 1:**
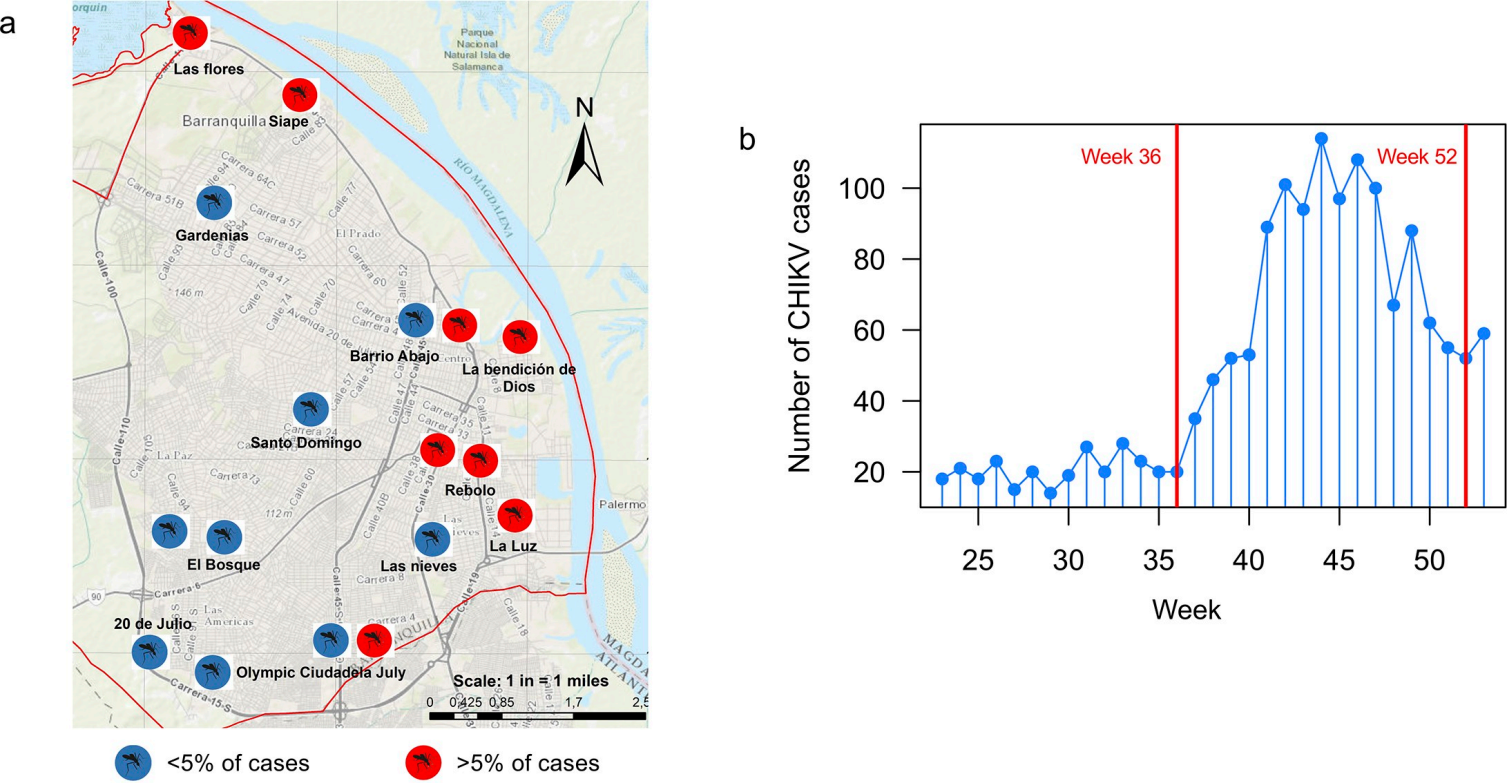
**(a)** Spatial distribution of 1160 patients, enrolled via health services in Barranquilla. All patients enrolled during the study agreed to disclose their address and were visited throughout the recruiting period. **(b)** Number of cases during the CHIKV outbreak in Barranquilla during weeks 36 to 52 of 2014. Cases included in this study were recruited during weeks 36 (September) to 52 (December) shown in red.

The city of Barranquilla, considered the main urban area in northern Colombia, is the capital of the Atlántico region and the fourth most populous city in the country with an estimated population of ~1.2 million inhabitants (Fig 1A).[26] Barranquilla has distinctive tropical weather conditions (average relative humidity of 80% and average temperature of 27°C). Strategically located next to the delta of the Magdalena river, the city serves as a port for river and maritime transportation within Colombia and is the main industrial, shopping, educational and cultural center of the Caribbean region of Colombia.

Patients were clinically diagnosed through face-to-face interview by the epidemiological surveillance team, following the WHO and CDC clinical evaluation recommendations.[25] The epidemiological surveillance team was formed of medical practitioners, nurses and health technicians who were responsible for gathering information about the CHIKV infectious disease emergency and carrying out case and contact investigations, in order to determine the epidemiological aspects of the CHIKV outbreak. The team collected information about suspected cases, possible contacts, disease characteristics, clinical characteristics, and possible disease exposure, in order to obtain, prioritize, and submit specimens for laboratory testing. Recruited CHIKV patients were in acute phases of the infection.

We actively collaborated with the Health Secretary Division of Barranquilla and its epidemiological surveillance team during the first and only CHIKV outbreak to date. The team received medical information about CHIKV clinically diagnosed patients from the main health providers (i.e., clinics and hospitals) in the city. These health providers were well-trained practitioners following the CHIKV diagnostic protocol recommended by the Colombian National Institute of Health (CNIH),[27] and constituted the first line of response against the infection. Patients reported by health providers were subsequently visited and surveyed by the epidemiological surveillance team.

### Ethics statement

The authors assert that all procedures contributing to this work have been performed in accordance with the ethical standards laid down in the 1964 Declaration of Helsinki and its later amendments.

### Subjects and case definition

Only clinically confirmed cases of CHIKV infection were included in this study. Diagnosis was assessed according to criteria described in CNIH guidelines[27] and reported during weeks 36 to 52 of 2014 (September to December; Fig 1B)—the peak period of the Chikungunya outbreak.[6]

Following the CHIKV protocol from the CNIH, patients were clinical diagnosed based on signs and symptoms, together with the epidemiological history of the population. Individuals were all residents in a geographical place where CHIKV presence was previously confirmed by laboratory testing (i.e., autochthonous cases). Signs and symptoms included fever >38°C, severe joint pain or initial acute arthritis symptoms and rash, which could not attributed to other medical conditions (and after discarding Dengue virus (DV) infection; S1 Table). Suspected cases were defined as residents of a geographical region where CHIKV presence had not been detected by laboratory testing. Signs and symptoms included fever >38°C, severe joint pain or initial acute arthritis symptoms and rash, could not be attributed to other medical conditions (and after discarding DV infection). Suspected cases were confirmed by laboratory testing using serum-based RT-PCR to detect viral RNA. Serum was sampled within the first eight days of the onset of symptoms. Patients presenting signs and symptoms characteristic of DV infection (S1 Table) were laboratory tested for IgM using ELISA. Those with positive tests for DV were excluded from this study.

### Clinical assessment

A total of 1322 individuals were interviewed and their clinical symptomatology subsequently registered. Since infection by CHIKV is a public health concern, and due to the Epidemiological Surveillance program being sanctioned by the Barranquilla Health Secretary, Colombian law allows the use of clinical material for research purposes without informed consent, which includes anonymous disclosure of results.

### Statistical analysis

#### Demographic characterization

Measures of central tendency and dispersion were estimated for continuous variables, and frequencies and proportions for categorical variables. Continuous variables among groups (gender) were compared using a two-sample *t* test when the normality assumption was met and the Wilcoxon-Mann-Whitney non-parametric test otherwise. The normality assumption was contrasted using the Shapiro-Wilks test. Frequencies and frequency distributions of categorical variables (i.e., gender and age group) among groups (i.e., gender) were compared using a χ^2^ test with continuity correction when the expected frequency of cells in the 2x2 contingency table was less than five. Logistic regression was used to correct for age when comparing these frequency distributions. Unless otherwise stated, statistical analyses and plotting were performed in R version 3.6.2 (R Foundation for Statistical Computing, Vienna, Austria, https://www.R-project.org). The False Discovery Rate (FDR)[28,29] was used to correct for multiple testing.

#### Characterization of CHIKV patients based on symptomatology

Based on the clinical symptoms, we derived clinical profiles using Latent Class Cluster Analysis (LCCA) [30] as implemented in Latent GOLD 4.0 (Statistical Innovations, Belmont, MA, USA). Symptoms were registered using a binary-based system assessing the presence or absence of 26 clinical symptoms in all patients of our cohort (0: absence; 1: presence), which were further used as indicators in all LCCA models tested. Models considering 1 to 10 clusters of individuals were explored including demographic information, such as sex and age, as covariates. In order to assess the certainty of our clusters, *P*-values associated with *L*^2^ statistics, by using a parametric bootstrap (500 replicates) rather than relying on asymptotic *P*-values, were estimated. Latent GOLD uses expectation/maximization and Newton-Raphson algorithms to find the maximum likelihood of each model after estimating model parameters. To avoid local solutions, which result from the use of maximum likelihood methods where a maximum is found that is not the true maximum of the entire sample space, a procedure automatically implemented in Latent GOLD was used.

## Results

### Participants

A total of 1322 subjects, who attended consultation at health providers, and were clinically diagnosed with CHIKV infection during the main CHIKV outbreak in the Caribbean region, were included in this study. Due to incomplete or missing data collection, 162 cases (12%) were excluded.

Our final sample consisted of 1160 patients (412 [35.5%] males, 748 [64.5%] females; Table 1). Of those, 129 (11.12%) were children (<15 years), 333 (28.7%) were adolescents or young adults (AYA; 15–29 years), 535 (46.12%) were adults (30–59 years) and 163 (14.05%) were 59 years or older (Table 1). Age ranged from 5 to 90 years old (mean age = 36.65, median age = 34, SD = 18.7). We found statistically significant differences in gender (*P*<0.0001; Table 1) and age group distributions (*P*<0.0001; Table 1). A significantly bigger proportion of CHIKV infected individuals inhabited low-income areas of the city, particularly areas near the border of the Magdalena river (east region)(Fig 1A). Main symptoms were fever (present in 94% of patients), headache (73%), general discomfort (59%), neck (54%) and back pain (53%), maculopapular rash (51%), myalgia (38%), arthritis (29%), conjunctivitis (7%), and nausea/vomiting (24%/21%) (Table 2). Some patients manifested to be suffering from persistent joint pain for months, even years.

**Table 1:** Demographic characteristics of patients included in this study.

Variable	Frequency (%)	χ^2^(df)	*P*
Gender			
Male	412 (35.52)	97.32 (1)	**<0.0001**
Female	748 (64.48)		
Age group		358.36 (3)	**<0.0001**
Children (<15)	129 (11.12%)		
AYA (15–29)	333 (28.70%)		
Adults (30–59)	535 (46.12%)		
Elderly (>59)	163 (14.05%)		

AYA: Adolescents and young adults; χ^2^: test statistic; *df*: degrees of freedom; *P*: *P*-value. Statistically significant results at 5% are shown in **bold**.

**Table 2:** Clinical symptomatology of CHIKV patients enrolled in this study.

Clinical Symptom	All (*n* = 1160; %)	Female (*n* = 748; %)	Male (*n* = 412; %)	χ^2^	***P***^*a*^
Fever	1095 (94.4)	705 (94.25)	390 (94.66)	0.024	0.852
Dizziness	328 (28.28)	226 (30.21)	102 (24.76)	3.636	**0.045**
Adynamia	119 (10.26)	80 (10.7)	39 (9.47)	0.313	0.469
Conjunctivitis	84 (7.24)	65 (8.69)	19 (4.61)	5.985	0.011*
Arthralgia	341 (29.4)	227 (30.35)	114 (27.67)	0.793	0.386
Difficulty to grasp	472 (40.69)	313 (41.84)	159 (38.59)	1.034	0.329
*Ganglia*					
Retroauricular	88 (7.59)	70 (9.36)	18 (4.37)	8.735	0.002*
Submandibular	66 (5.69)	55 (7.35)	11 (2.67)	10.003	0.002*
Cervical	43 (3.71)	35 (4.68)	8 (1.94)	4.837	**0.022**
Axillary	55 (4.74)	45 (6.02)	10 (2.43)	6.802	0.009*
Inguinal	65 (5.6)	52 (6.95)	13 (3.16)	6.539	0.009*
General discomfort	689 (59.4)	451 (60.29)	238 (57.77)	0.603	0.356
Nausea	284 (24.48)	200 (26.74)	84 (20.39)	5.455	0.012*
Cough	328 (28.28)	222 (29.68)	106 (25.73)	1.855	0.176
Headache	850 (73.28)	560 (74.87)	290 (70.39)	2.497	0.092
Vomit	247 (21.29)	178 (23.8)	69 (16.75)	7.462	0.002*
Pruritus	310 (26.72)	206 (27.54)	104 (25.24)	0.604	0.369
Myalgias	444 (38.28)	282 (37.7)	162 (39.32)	0.230	0.573
Skin sensitivity	301 (25.95)	206 (27.54)	95 (23.06)	2.549	0.123
Feverish chill	594 (51.21)	389 (52.01)	205 (49.76)	0.451	0.500
Lack of appetite	447 (38.53)	303 (40.51)	144 (34.95)	3.233	0.084
*Pain*					
Abdominal	435 (37.5)	289 (38.64)	146 (35.44)	1.028	0.264
Calf	592 (51.03)	399 (53.34)	193 (46.84)	4.232	0.052
Neck	632 (54.48)	427 (57.09)	205 (49.76)	5.461	**0.034**
Back	614 (52.93)	408 (54.55)	206 (50)	2.025	0.180
Hip	426 (36.72)	312 (41.71)	114 (27.67)	21.940	**7.43x10**^-6^*

^*a*^ Corrected for age using Logistic Regression. χ^2^: test statistic; *P*: *P*-value. Statistically significant results at the 5% nominal level are shown in **bold**, and those significant after correction for multiple testing correction using FDR are highlighted with *.

### Symptomatology in patients with CHIKV

**Fig 2:**
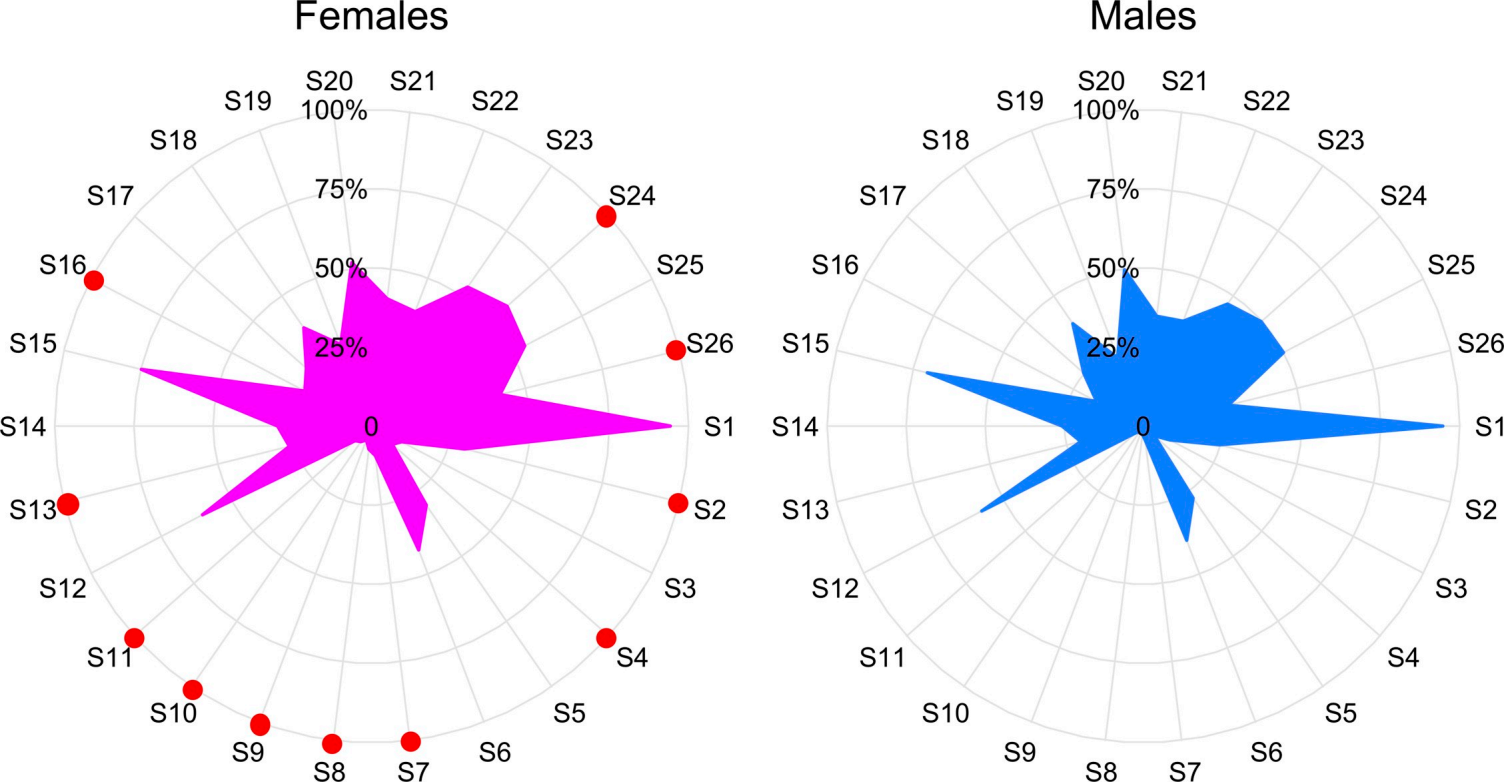
Radial plots by gender displaying the percentage of individuals exhibiting each clinical symptom. Symptoms with red dots are more frequently present in females than in males (Table 2). S1: Fever, S2: Dizziness, S3: Adynamia, S4: Conjunctivitis, S5: Arthralgia, S6: Difficulty grasping, S7: Retro auricular ganglia, S8: Submandibular ganglia, S9: Cervical ganglia, S10: Axillary ganglia, S11: Inguinal ganglia, S12: General discomfort, S13: Nausea, S14: Cough, S15: Headache, S16: Vomiting, S17: Pruritus, S18: Myalgias, S19: Skin sensitivity, S20: Feverish chill, S21: Lack of appetite, S22: Abdominal pain, S23: Calf pain, S24: Neck pain, S25: Back pain, S26: Hip pain.

We clinically assessed 26 symptoms in our study cohort of CHIKV patients (Table 2). Individuals showed a myriad of symptoms including fever, dizziness, pruritus and myalgia, as well as different types of pain. Of all the symptoms, fever (94.4%, *n* = 1,095), headache (73.28%, *n* = 850) and general discomfort (59.4%, *n* = 689) were the most frequent, while inguinal (5.6%, *n* = 65), axillary (4.74%, *n* = 55), and cervical (3.71%, *n* = 43) ganglia were the least frequent (Table 2). Interestingly, women reported higher occurrence of dizziness (30.2% women vs. 25% men; *P* = 0.045), conjunctivitis (9% women vs. 4.6% men; *P* = 0.011), localized adenopathy (retroauricular, submandibular, axillary; *P*<0.05), nausea (26.7% women vs. 20.4% men; *P* = 0.012), vomiting (23.8% women vs. 16.8% men; *P* = 0.002), and neck (57.1% women vs. 49.8% men; *P* = 0.034) and hip pain (41.1% women vs. 27.6% men; *P*<0.0001)(Fig 2 and Table 2).

### Symptomatology profiles in CHIKV infected patients

**Fig 3:**
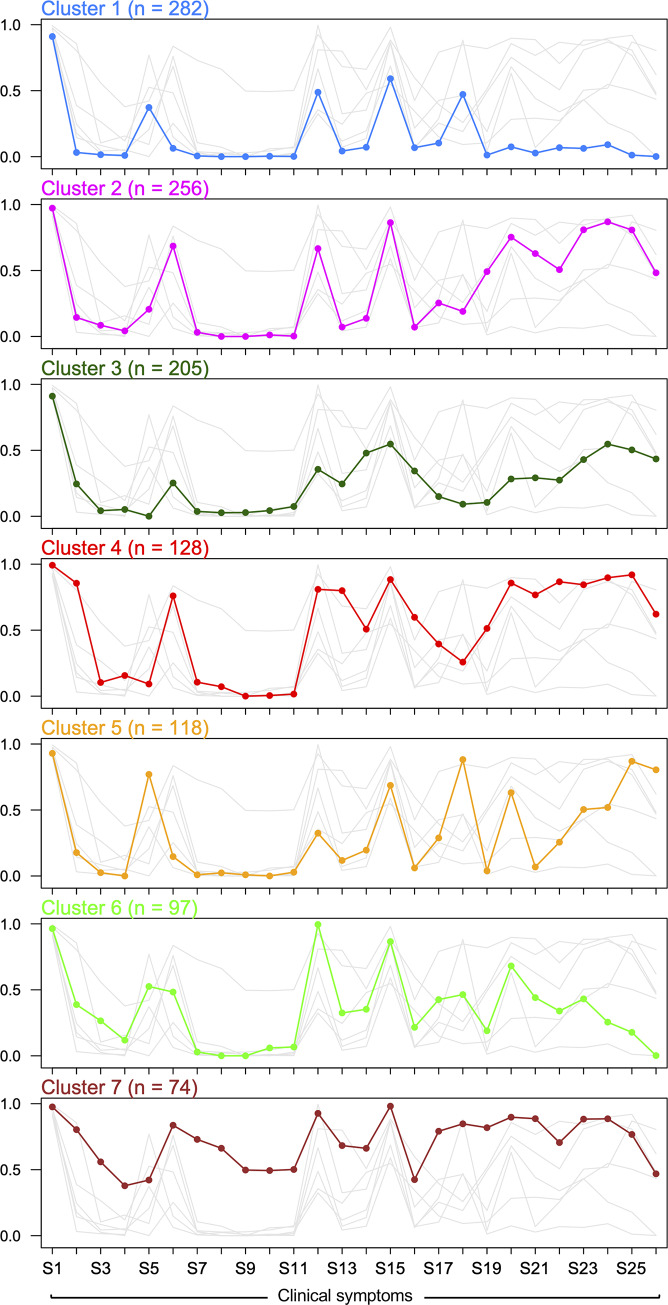
Profile plot of the LCCA-derived clusters. Here, the *x*-axis represents each of the CHIKV symptoms, and the *y*-axis represents the probability of having each clinical symptom. Conventions as in Fig 2.

We identified seven mutually exclusive latent clusters of individuals exhibiting differential and statistically significant symptom profiles, suggesting the existence of distinct subpopulations within patients with CHIKV infection (Fig 3 and Table 3). Of the 1061 patients, 282 (24.3%) were assigned to Cluster 1, 256 (22.1%) to Cluster 2, 205 (17.7%) to Cluster 3, 128 (11%) to Cluster 4, 118 (10.2%) to Cluster 5, 97 (8.4%) to Cluster 6 and 74 (6.4%) to Cluster 7. Analysis showed that the number of symptoms is statistically significantly different across clusters (*F*_6,1151_ = 744.5, *P*<0.00001; S1 Fig).

**Table 3:** Summary of LCCA-derived clusters in individuals with CHIKV infection.

Clinical Symptom	Cluster 1	Cluster 2	Cluster 3	Cluster 4	Cluster 5	Cluster 6	Cluster 7
	*n* (%)	*n* (%)	*n* (%)	*n* (%)	*n* (%)	*n* (%)	*n* (%)
	282 (24.3)	256 (22.1)	205 (17.7)	128 (11)	118 (10.2)	97 (8.4)	74 (6.4)
** **							
Fever	**256 (90.8)**	**249 (97.3)**	**187 (91.2)**	**127 (99.2)**	**110 (93.2)**	**94 (96.9)**	**72 (97.2)**
Dizziness	10 (3.5)	33 (12.9)	52 (25.4)	**113 (88.3)**	23 (19.5)	39 (40.2)	**60 (80.6)**
Adynamia	4 (1.4)	22 (8.6)	9 (4.4)	12 (9.4)	3 (2.5)	27 (27.8)	**42 (56.9)**
Conjunctivitis	2 (0.7)	11 (4.3)	11 (5.4)	20 (15.6)	0 (0)	13 (13.4)	28 (37.5)
Arthralgia	103 (36.5)	50 (19.5)	0 (0)	10 (7.8)	**93 (78.8)**	**53 (54.6)**	32 (43.1)
Difficulty to grasp	21 (7.4)	**182 (71.1)**	45 (21.9)	**98 (76.6)**	16 (13.6)	**49 (50.5)**	**62 (83.3)**
*Ganglia*							
Retroauricular	1 (0.4)	8 (3.1)	8 (3.9)	14 (10.9)	1 (0.8)	3 (3.1)	**54 (73.6)**
Submandibular	0 (0)	0 (0)	6 (2.9)	9 (7)	3 (2.5)	0 (0)	**49 (66.7)**
Cervical	0 (0)	0 (0)	6 (2.9)	0 (0)	1 (0.8)	0 (0)	37 (50)
Axillary	1 (0.4)	3 (1.2)	9 (4.4)	0 (0)	0 (0)	6 (6.2)	37 (50)
Inguinal	0 (0)	1 (0.4)	16 (7.8)	2 (1.6)	3 (2.5)	7 (7.2)	37 (50)
General discomfort	140 (49.6)	**174 (68)**	70 (34.2)	**102 (79.7)**	38 (32.2)	**97 (100)**	**69 (93.1)**
Nausea	11 (3.9)	13 (5.1)	57 (27.8)	**106 (82.8)**	14 (11.9)	33 (34)	**50 (68.1)**
Cough	18 (6.4)	27 (10.5)	**105 (51.2)**	68 (53.1)	24 (20.3)	37 (38.1)	**50 (68.1)**
Headache	**169 (59.9)**	**221 (86.3)**	**107 (52.2)**	**114 (89.1)**	**83 (70.3)**	**85 (87.6)**	**73 (98.6)**
Vomit	19 (6.7)	14 (5.5)	76 (37.1)	**80 (62.5)**	6 (5.1)	22 (22.7)	31 (41.7)
Pruritus	28 (9.9)	68 (26.6)	32 (15.6)	48 (37.5)	33 (28)	43 (44.3)	**60 (80.6)**
Myalgias	130 (46.1)	46 (18)	17 (8.3)	32 (25)	**107 (90.7)**	**49 (50.5)**	**63 (84.7)**
Skin sensitivity	3 (1.1)	131 (51.2)	20 (9.8)	67 (52.3)	4 (3.4)	17 (17.5)	**61 (81.9)**
Feverish chill	20 (7.1)	**191 (74.6)**	60 (29.3)	**111 (86.7)**	**76 (64.4)**	**69 (71.1)**	**67 (90.3)**
Lack of appetite	7 (2.5)	**163 (63.7)**	60 (29.3)	99 (77.3)	7 (5.9)	45 (46.4)	**67 (90.3)**
*Pain*							
Abdominal	22 (7.8)	**135 (52.7)**	50 (24.4)	**113 (88.3)**	30 (25.4)	35 (36.1)	**51 (69.4)**
Calf	17 (6)	**207 (80.9)**	89 (43.4)	**111 (86.7)**	**60 (50.8)**	43 (44.3)	**66 (88.9)**
Neck	27 (9.6)	**224 (87.5)**	**115 (56.1)**	**117 (91.4)**	**62 (52.5)**	21 (21.6)	**66 (88.9)**
Back	2 (0.7)	**208 (81.2)**	**108 (52.7)**	**118 (92.2)**	**104 (88.1)**	16 (16.5)	**58 (77.8)**
Hip	0 (0)	123 (48)	94 (45.9)	80 (62.5)	**96 (81.4)**	0 (0)	34 (45.8)
Sex							
Female	**166 (58.8)**	**163 (63.7)**	**146 (71)**	**92 (71.9)**	**70 (59.4)**	**51 (52.5)**	**61 (82.9)**
Male	116 (41.2)	93 (36.3)	59 (29)	36 (28.1)	48 (40.6)	46 (47.5)	13 (17.1)
Age group							
Children	40 (14.2)	23 (9)	18 (8.8)	15 (11.7)	11 (9.3)	18 (18.6)	4 (5.4)
AYA	89 (31.6)	56 (21.9)	55 (26.8)	40 (31.2)	33 (28)	**38 (39.2)**	22 (29.7)
Adults	**122 (43.3)**	**128 (50)**	**95 (46.3)**	**64 (50)**	**56 (47.5)**	32 (33)	**38 (51.4)**
Elderly	31 (11)	49 (19.1)	37 (18)	9 (7)	18 (15.3)	9 (9.3)	10 (13.5)

AYA: Adolescents and young adults. LCCA: Latent class cluster analysis. Numbers in **bold** correspond to symptoms, sex category or age group more likely to be present by cluster.

**Fig 4:**
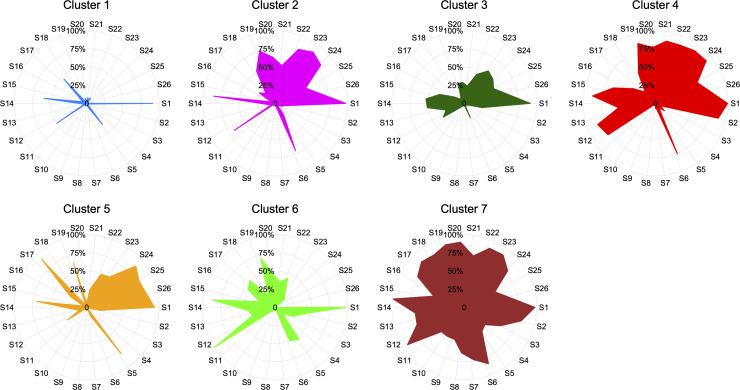
Radial plots displaying the probability of CHIKV symptoms for each cluster derived using latent class cluster analysis. Conventions as in Fig 2.

Based on this symptom profiling, Cluster 1 outlines a subgroup of individuals (*n* = 282, 24.3%) with minor symptomatology (women with fever and headache as unique manifestations; Table 3), while individuals in Cluster 2 (*n* = 256, 22.1%) exhibit fever, difficulty grasping, general discomfort, headache, skin sensitivity, feverish chill, lack of appetite, and abdominal, calf, neck and back pain (Table 3). On the other hand, Cluster 3 is comprised of individuals (*n =* 205, 17.7%) who are more likely to experience fever, cough, headache, and neck and back pain, while Cluster 4 (*n* = 128, 11%) groups individuals with a symptomatology profile characterized by fever, dizziness, difficulty grasping, general discomfort, nausea, cough, headache, vomiting, skin sensitivity, feverish chill, lack of appetite, as well as abdominal calf, neck, back and hip pain. Individuals belonging to Cluster 5 (*n* = 118, 10.2%) have a symptomatology mainly characterized by fever, arthralgia, headache, myalgias and calf, neck, back and hip pain. In contrast, individuals in Cluster 6 (*n* = 97, 8.4%) are more likely to experience fever, arthralgia, difficulty grasping, general discomfort, headache, myalgias and feverish chill. Finally, Cluster 7 gathers individuals (*n* = 74, 6.4%) with complex symptomatology (fever, dizziness, adynamia, difficulty grasping, retroauricular and submandibular ganglia, general discomfort, nausea, cough, headache, pruritus, myalgias, skin sensitivity, feverish chill, lack of appetite, and diverse sorts of pain) (Fig 3 and Table 3). Individuals belonging to Clusters 1 and 7 represent extreme profiles of phenotypic response as they deviate from the natural history of the disease (Figs 3 and 4), and confirm the divergence of the clinical symptomatology spectrum. Interestingly, all LCCA-derived clusters are mostly constituted of females, who, as previously discussed, show a differential phenotypic response pattern when compared to men (Fig 2 and Table 2). The significance of the χ^2^-based test of association for gender (χ^2^ = 34.41, degrees of freedom [*df*] = 6, *P*<0.0001), age group (χ^2^ = 42.03, *df* = 18, *P* = 0.001) and gender and age group (χ^2^ = 84.16, *df* = 39, *P*<0.0001) by cluster, showed that these variables are both independently and jointly associated with a differential phenotypic response in individuals with CHIKV infection. Closer inspection of the characteristics of the LCCA-derived clusters revealed that the Female:Male ratio by age group is higher in clusters 4 and 7, particularly in the AYA and adult groups, although the extreme patterns of phenotypic response occur in the latter age group for cluster 7.

## Discussion

The overarching hypothesis tested by this study (i.e., the existence of significantly different latent classes of symptoms, clustering individuals infected by CHIKV during the highest outbreak in Barranquilla) was not rejected. Indeed, we found seven different symptom profiles clustering groups of individuals with distinguishable phenotypic response. Thus, this could potentially be used for defining extreme and intermediate phenotypes of CHIKV infection. Furthermore, the fact that *i*) females show distinct phenotypic response pattern compared to men (Fig 2 and Table 2), *ii*) all LCCA-derived clusters are mostly comprised by females (Figs 3 and 4, and Table 3), and *iii*) that there is a relationship between the number of symptoms and the cluster of CHIKV infected individuals (S1 Fig), highlights the importance of gender-specific and phenotypic-response-specific treatments focused on the at-risk female CHIKV population, and subpopulations with a more extreme phenotypic spectrum (i.e., clusters 1 and 7; Fig 3), instead of a one-size-fits-all approach.[31,32] A more refined characterization of CHIKV patient sub-populations (LCCA-derived clusters) may be advantageous for the implementation of specific diagnosis protocols and treatments, which could allow health providers to implement more personalized and cost-effective care for CHIKV patients, depending on their phenotypic response to the infection.

Sex dimorphic immune response towards viral infection is commonly described in the literature.[33] For example, it is well known that women respond with production of higher levels of Interferon alpha (INFα) in response to viral pathogens.[33,34] Furthermore, sex dimorphic immune response to viral infections has shown higher intensity (i.e. viral load within an individual) and prevalence (i.e. number of infected individuals within a population) in males, while females can have a more favorable disease outcome.[35] Females have a stronger immune response relative to males, which can result in faster viral clearance. *In vitro* experiments have shown that cells from females can exhibit a 10‐fold greater level of expression than cells from males.[35] However, this may also contribute to the development of autoimmunological disorders in females, such as, systemic lupus erythematosus, Graves’ disease, Hashimoto’s thyroiditis, multiple sclerosis, rheumatoid arthritis and scleroderma.[36]

A stronger female immune response towards CHIKV infection may be associated with a complex symptomatology (fever, dizziness, adynamia, difficulty grasping, retroauricular and submandibular ganglia, general discomfort, nausea, cough, headache, pruritus, myalgias, skin sensitivity, feverish chill, lack of appetite, and diverse sorts of pain) for women compared to men in our study. Epidemiological studies have shown that males are more vulnerable to viral infections as their mortality rate is higher compared to women,[37] and women tend to have a more efficient humoral and cellular immune response against viruses.[35,38] Although a stronger immune response is clearly an advantage for certain viral infections, with CHIKV infection a potential dissemination of viral particles to lymph nodes, joints and other tissues may lead to aberrant antigenic response that could worsen the symptomatology in women as compared to men.[39–41] Kam et al. [42] investigated the CHIKV route of infection starting at epithelial and endothelial cells, primary fibroblasts, monocytes and monocyte-derived macrophages, further disseminating to lymph nodes, joints and other tissues.[43] Spread of the infection to the joints results in arthralgia, which mirrors rheumatoid arthritis, a condition characterized by joint pain as the result of tissue inflammation and destruction by inflammatory cytokines such as IL-1β, IL-6 and TNF-α.[44]

We identified different clinical signatures in individuals with CHIKV (Fig 2 and Table 3). For instance, cluster 2 only presented 11/26 (42.3%) symptoms with probability above 50%, while cluster 7 exhibited 24/26 (92.3%) symptoms. Furthermore, sexual dimorphism is not as differentiable in clusters 2, 5 and 6, while there was a distinctive dimorphism in clusters 3, 4 and 7 (Fig 2 and Table 3). Although we demonstrate distinct CHIKV phenotypic response subpopulations, there are different factors that have shown to modulate human response to arbovirus infection and hence be responsible for the clinical symptomatology differentiation presented herein. Some of these factors include (*i*) vector competence, that is, the ability of the vector to acquire the virus and successfully transmit it to a susceptible host;[45] (*ii*) the variety of the viral strain, in which genetic studies have shown that CHIKV has evolved into three distinct genotypes—west African, East/Central/South African, and Asian;[46] (*iii*) environmental conditions such as ecological factors, global population growth, urbanization, lack of mosquito control measures and decay in public health;[23] and (*iv*) population genetics, that is, human individual differences such as genetic makeup and single nucleotide polymorphisms (SNPs), mostly associated with immunological pathways.[23] Indeed, genetic markers rs179010, rs5741880 and rs3853839 in the *TLR-7* gene, and rs3764879 in the *TLR-8* gene have been associated with increased CHIKV infection susceptibility.[47] These SNPs were also found to be associated with enhanced susceptibility of patients infected with CHIKV to developing fever, joint pain, and rashes.[47] As our study population has a strong African admixture,[48–50] [51] future studies should focus on determining the contribution of these SNPs in this understudied population. Furthermore, considering that CHIKV originated in the African continent, genetic ancestry in our population could help to elucidate its influence on host response. This will be crucial for developing monitoring and diagnostic tools as well as more accurate diagnosis and treatment options, and for outlining public health policies.

According to the epidemiologic surveillance office, the east and northeast areas of Barranquilla and its metropolitan area (Fig 1A) are the most severely impacted during arthropod virus outbreaks, such as dengue, an endemic disease in the city. Although in the present study we only included clinically confirmed cases of CHIKV infection, all cases were defined according to the criteria described by the Guidelines of the Colombian National Institute of Health,[27] and no other outbreak related to other arboviruses in Barranquilla during the study period was reported. In this regard, the CNIH reported 104,389 clinically confirmed cases and 1410 laboratory confirmed cases in 2014,[52,53] with estimates of asymptomatic CHIKV infection varying around ~3–25%.[54] Nevertheless, unlike other arboviruses, the quantity of asymptomatic CHIKV infection patients is low. In addition, experience of the surveillance of arboviruses in Colombia is robust and has generated data of adequate reliability.

Co-occurrence of two or more symptoms may influence symptom recognition and harmfully affect individuals’ quality of life, negatively impacting individuals’ health condition, and accelerating the timing of clinical treatment. Finding that two or more symptoms co-occur or happen in particular combinations in individuals infected with CHIKV during the major outbreak in the Colombian Caribbean region may potentially help to identify specific symptom patterns more clearly in CHIKV infection and facilitate patient management. Future studies assessing the contribution of demographic, immunological and genetic factors to symptom co-occurrence, and correlating this symptomatology with viral strain and/or CHIKV genetic variation could shed some light on the severity of the clinical symptomatology and, ultimately, lead to more accurate, more efficient and differential diagnosis. It will be fascinating to revisit this cohort as new advances in diagnostic and next generation sequencing techniques become more accessible.

Despite our new findings, some limitations of our study must be acknowledged. First, our maximally expanded sample was comprised of 1161 individuals diagnosed with CHIKV infection based on clinical symptomatology. Of note, individuals were recruited during the acute phase of the CHIKV infection. This diagnosis was performed following a clinical protocol developed by the Colombian National Institute of Health based on a similar protocol previously applied to diagnosis of Dengue virus infection (S1 Table). The chikungunya outbreak occurred in the Colombian Caribbean back in 2014/15, which also impacted the Central/Andean region of the country. This protocol was strictly followed by the epidemiological team of the Health Secretary of Barranquilla to survey all suspected CHIKV cases referred to the surveillance program by health practitioners from the city of Barranquilla. To confirm the feasibility of the clinical protocol, and to detect the presence of CHIKV, we randomly selected 300 (25.9%) of our CHIKV cases and conducted laboratory testing using RT-PCR. Interestingly, we found concordance between the clinical-symptomatology-based CHIKV infection diagnosis and that provided by the laboratory test. All in all, this suggests that, despite not having laboratory tested all individuals in our sample, the clinical diagnosis, when applied responsibly, is a reliable and easy-to-use method of providing accurate diagnosis of CHIKV infection in developing and Latin American countries, where budget and time constraints are increasing, or during outbreak episodes such as the 2014 CHIKV outbreak in the Colombian Caribbean region.

A second limitation of our study is the lack of outcome data, such as vital signs, duration of symptoms and disease evolution. Because of this, we were unable to establish correlations between such variables and the profiles of phenotypic response exhibited by individuals with CHIKV infection (Fig 3 and Fig 4). Although we identified that the number of clinical symptoms is associated with the cluster CHIKV infected individuals belong to (S1 Fig), more research studies are needed, especially those of a longitudinal nature, to better understand long-term outcomes in individuals with CHIKV infection exhibiting such patterns of phenotypic response to the virus. We argue that these studies will greatly benefit from our findings. In fact, such studies could eventually use the differences between males and females in terms of clinical symptomatology (Fig 2 and Table 2), as well as the subpopulations we identified (Figs 3 and 4, and Table 3), to identify at-risk populations or use multi-omics approaches to detangle the genetics, epigenetics and proteomics underpinning distinct complex phenotypic (extreme) responses in these individuals (i.e., clusters 1 and 7; Figs 3 and 4).

In summary, our study provides, for the first time, strong evidence supporting the existence of different subpopulations among individuals infected with CHIKV, including important differences in patterns of phenotypic response between males and females, which highlights the importance of developing gender-specific approaches to treating this infection. In low-, mid-income and Latin American countries, the use of these characterizations could enhance the development, validation and implementation of alternative ways of diagnosing CHIKV infection based on clinical symptomatology machine learning (ML) algorithms, to accurately identify such patterns in a timely fashion in the clinical setting,[55] in order to provide personalized treatment.[56] ML algorithms have recently proven to be effective, accurate and easy to use in CHIKV,[57] and other infectious diseases.[58] Preliminary results of the implementation of ML algorithms using our set of patients yielded a maximum correct classification rate (i.e., accuracy) of 91.9% (S2 Table).

## Supporting information

S1 Fig Beanplots for the number of symptoms in individuals diagnosed with CHICKV in Barranquilla, Colombia, by LCCA cluster. ANOVA analysis shows that the number of symptoms differs by cluster (*F*_6,1151_ = 744.5, *P*<0.00001). In particular, individuals in clusters 1 and 7 differ substantially. As mentioned in the Main text, these clusters are of special interest as represent extreme clinical profiles (i.e., phenotypic expression/symptomatology).
(TIF)

S1 Table Differential diagnosis between CHIKV and Dengue virus. (XLSX)

S2 Table Accuracy of different Machine Learning algorithms to predict differential phenotypic response (i.e., cluster) in individuals with CHIKV infection using clinical symptomatology. Algorithms were implemented in R. Here, the accuracy corresponds percentage of individuals correctly classified in the testing data set (*n* = 344).
(XLSX)
