# Supplementary figures and images for: Chikungunya outbreak (2015) in the Colombian Caribbean: Latent classes and gender differences in virus infection
Source: PLoS Negl Trop Dis. 2020 Jun 3;14(6):e0008281. doi: 10.1371/journal.pntd.0008281 (PMC7304630; doi:10.1371/journal.pntd.0008281)

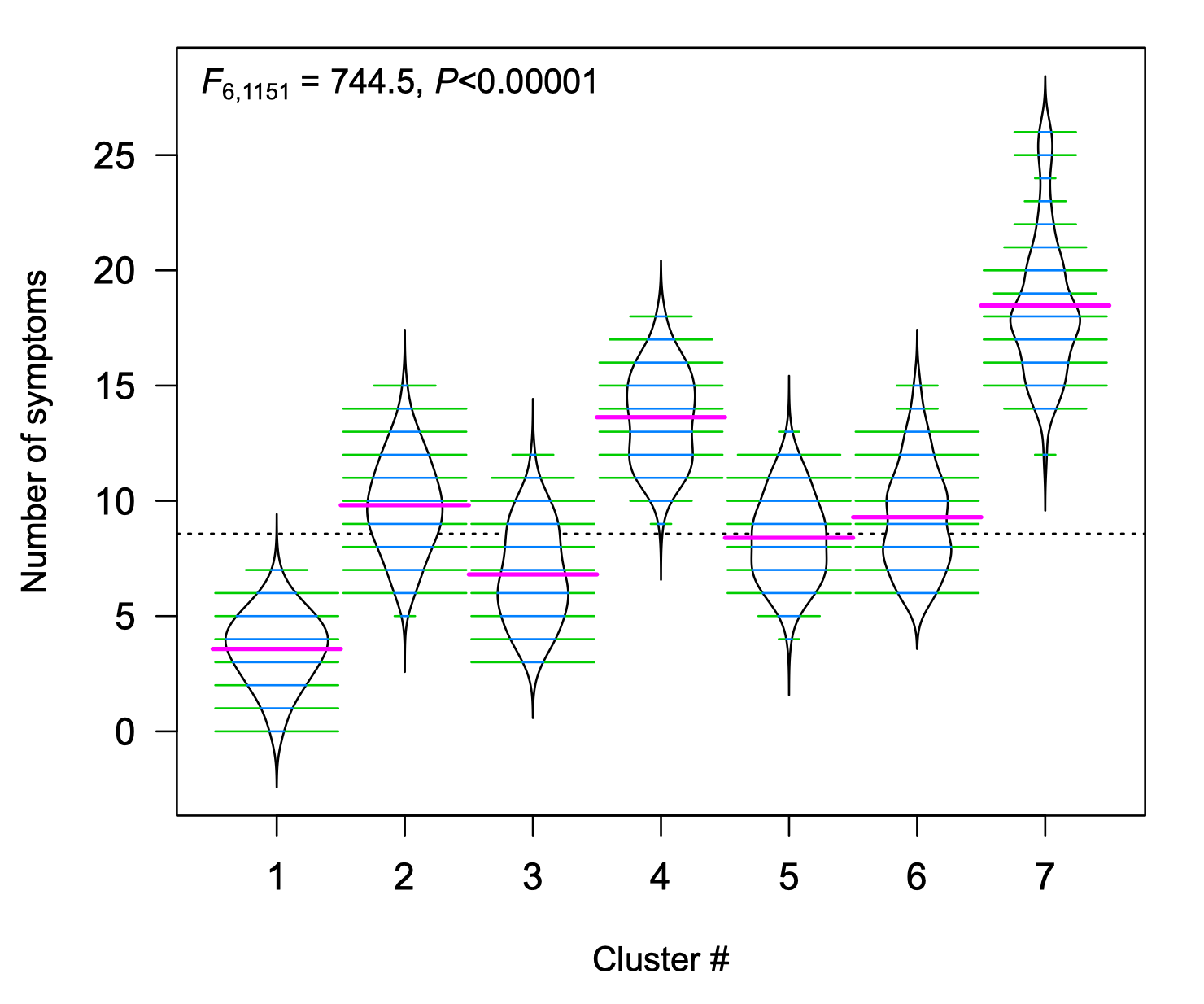

Supplement: S1 Fig — ANOVA analysis shows that the number of symptoms differs by cluster (F6,1151 = 744.5, P<0.00001). In particular, individuals in clusters 1 and 7 differ substantially. As mentioned in the Main text, these clusters are of special interest as represent extreme clinical profiles (i.e., phenotypic expression/symptomatology). (TIF) [file pntd.0008281.s001.tif]
